# Reliability of self, parental, and researcher measurements of head circumference

**DOI:** 10.1186/2040-2392-5-2

**Published:** 2014-01-10

**Authors:** Jillian C Sullivan, Teresa Tavassoli, Kimberly Armstrong, Simon Baron-Cohen, Ayla Humphrey

**Affiliations:** 1 Department of Psychiatry, Autism Research Centre, University of Cambridge, Cambridge, UK; 2 Autism Research Centre, Douglas House, Trumpington Road, Cambridge CB2 8AL, UK

**Keywords:** Adults, Brain size, Children, Head circumference, Reliability, Self-measurements, Parental measurements

## Abstract

**Background:**

The measurement of head circumference (HC) is widely used in clinical and research settings as a proxy of neural growth. Although it could aid data collection, no studies have explored either the reliability of adult self-measurements or parental measurements of young children. This study therefore aimed to examine whether adult self and parental measurement of HC constitute reliable data.

**Findings:**

A total of 57 adults (32 male) were asked to measure their HC twice following written instructions (adult self-measurement). These measures were compared to those of a researcher independently measuring the same participant’s HC twice. Additionally, mothers of 25 children (17 male) were also asked to measure their child’s HC (parental measure), and again this was compared to researcher measurements of the child’s HC. The intraclass correlation coefficient between adult self- and researcher measurement was 0.84 and between parent and researcher measurement was 0.99. The technical error of measurement was also acceptable, within the range of a skilled anthropometrist.

**Conclusions:**

The high degree of agreement between researcher and adult self-measurement/parental measurement of HC demonstrates that these different assessors produce similarly reliable and reproducible data. This suggests adult self- and parental measurements can reliably be used for data collection to enable valid large-scale developmental and clinical studies of HC.

## Background

Head circumference (HC) is a widely used proxy of neural growth and brain size in paediatric and research settings [1]. HC, as the occipital-frontal perimeter of the head, is related to individual differences in neuroanatomy [2]. It has numerous correlates in the typical and clinical population such as cognitive ability [3] and both risk and outcome for a number of neurological and genetic conditions [4]. Abnormal HC growth trajectories have also been reported in children and adults in a number of mental health and high risk populations, including autism [5], schizophrenia [6], dementia [7], premature birth [8], and malnourishment or global privation [9,10]. Findings in the autism population suggest that abnormal brain growth may in fact play an important role in the aetiology and progression of the disorder [11], although there has recently been some concerns about HC norms and robustness of measurement methodology in this population as well as in the comparison of brain volume to HC in the general population [12,13].

Larger samples and longitudinal data would enable a better understanding of this phenomenon. As HC is a relatively quick and cost-effective assessment and can provide information on risk for some medical and neurological problems, it would be useful to establish if the general population is as accurate as trained professionals in producing these measurements. Indeed, HC measurements are often considered difficult even by professionals due to individual differences in head shape, hair styles and texture, and subject cooperation as well as examiner differences in tape measure placement and tautness. Moreover, the authors are unaware of standardized guidelines for measuring HC and different agencies recommend different tape measure tools and techniques (e.g., [14]), although there appears to be consensus that the tape measure must be pulled snugly and that the maximum distance around the head should be recorded. To this end, the current study investigated whether (a) adults can reliably measure their own HC (adult self-measure), and (b) whether parents can reliably measure their child’s HC (parental measure). No such reliability study has been performed on these populations, although Bradley et al. [15] did find that parental measurements of the HC of 1- to 6-week-old infants were reliable enough for individual-level analysis according to intraclass correlation coefficients (ICC). There is, however, a need for a reliability study of parents of children older than 6 weeks of age as well as on adult self-measurements of HC. Moreover, with anthropometric measurements, technical error of measurements (TEM), the standard deviation of the difference between repeated measurements, should be calculated rather than ICC, which does not account for bias in measurements [16].

## Methods

### Participants

In total, 57 adults (32 male, 18–48 years old) in the Cambridge area were targeted via an opportunistic sampling method. A second opportunistic sample included 25 mothers of typically developing children (17 male; 9 months to 7 years) who were asked to measure their child’s HC.

### Procedure

Participants were given a measuring tape and written instructions/diagram explaining the HC measurement: the instructions included a photograph of an adult male measuring his own HC, and asked the participant to measure their head above the ears and slightly above the eyebrows in order to capture the maximum distance around their head, and to pull the tape measure tightly. It was also emphasized that HC differences between individuals are small and that accuracy and detailed measurements (e.g., using half centimetres if appropriate) were essential (see Additional file 1). Participants were asked to record measurements from their own head or their child’s head twice without assistance. Researchers gave no verbal instructions on HC measurements nor offered advice. Subsequently, a trained researcher, blind to participant’s measurements, measured the participant’s head twice, as did a second trained researcher in subsample of the adult participants (n = 44). Non-stretchable fiberglass tape measures were used in order to conform to the type of tape measure that participants would most likely have at home. All participants signed consent forms and these studies received ethical approval from the Cambridge Psychology Research Ethics Committee.

## Results

Two versions of TEM were calculated, as detailed in Perini et al. [17]. Absolute TEM was calculated as $\left(\sqrt{\sum d^{2}}\right)/2n$, where ∑ *d*^2^ is the sum of the difference in measurements squared for each participant and *n* is the number of participants in the sample. Next, relative TEM was calculated from the absolute TEM to illustrate the error in HC measurement as a percentage of the average HC measurement by dividing the absolute TEM by the grand mean of individual measurement averages and multiplying it by 100. Studies have shown that acceptable relative TEM percentages for within-rater measurements are below 1.5% for beginner anthropometrists and below 1.0% for skilled anthropometrists, while acceptable limits for between-rater measurements are 2.0 and 1.5%, respectively [17]. For comparison to previous studies, ICCs (two-way, mixed measure, absolute agreement) were also calculated.

Within-rater reliability was explored as a function of the two HC measurements taken by the adult self-measurements, the parental measurements, and each of the two researcher’s HC measurements of the adult or child. Between-rater reliability was assessed as the concordance between the mean HC measured by (a) the adult-self or parental-child and the primary researcher, and (b) researcher 1 and researcher 2 measuring the adult’s HC.

### Within-rater reliability

Separate absolute and relative TEM was calculated for the differences in the two HC measurements for the participant’s adult self- or parental measurements (n = 57 adults, n = 25 children), researcher A (n = 57 adults, n = 25 children), and researcher B (n = 47 adults). All relative TEM were below 1.0 and within the acceptable range for a skilled anthropometrist, with the TEM for the mothers measuring their child’s HC being especially low in error.

Adult self-measurement intra-rater reliability, according to ICC calculations, was high, with the participants showing an ICC = 0.94, (95% confidence interval (CI) 0.90–0.96) between their two self-measurements. Researchers showed similarly high self-reproducibility, between their own two measurements of adults: Researcher A, ICC = 0.98 (95% CI: 0.97–0.99); Researcher B, ICC = 0.98 (95% CI: 0.96–0.99). The researchers measuring the children’s heads were also very reliable, with an ICC = 0.99 (n = 25; 95% CI: 0.99–1.00) between their two measurements. Parental measures of their child’s head were very reliable (ICC = 0.99; n = 25; 95% CI: 0.98–0.99). The 95% CI for the researcher ICC and the participant ICC overlapped; consequently, reliability estimates could not be statistically differentiated. In other words, self-measurements were as reliable as researcher measurements.

### Between-rater reliability

To calculate between-rater TEM, two analyses were conducted. The first may be considered to be an index of self-/parent-measurement validity as the difference between the mean of the two self-/parent-measurements were compared with the mean of researcher A’s two measurements. Again, the between-rater relative TEM was in the acceptable range for a skilled anthropometrist for both adult self- and parental measurements (see Table 1 for all TEM results). The ICC between researcher A’s mean measurement and the adult’s mean measurement (n = 57) was also acceptable (ICC = 0.84; 95% CI: 0.74–0.90), as was the ICC between parental and researcher mean HC (ICC = 0.99; 95% CI: 0.99–1.00; n = 25).

**Table 1:** **Absolute and Relative Technical Error of Measurement** (**TEM**) **for adult self**-**measurement**, **parental measurement**, **and researcher measurement of head circumference**

	**Adult self-measurement**		**Parent measurement of child**	
	**Absolute TEM**	**Relative TEM**	**Absolute TEM**	**Relative TEM**
**Within-rater**				
Adult self/parental	0.52	0.91%	0.29	0.57%
Researcher A	0.26	0.45%	0.16	0.31%
Researcher B	0.31	0.53%	--	--
**Between-rater**				
Self/child vs. researcher A	0.82	1.43%	0.20	0.39%
Both researchers	0.45	0.78%	--	--

Secondly, between-researcher TEM and ICC were also good for the adult sample (n = 47), with the TEM for the difference between researcher mean measurements falling within the acceptable limits for a skilled anthropometrist and the ICC = 0.93 (95% CI: 0.88–0.96).

## Discussion

The results of this study show high levels of reliability of HC by adult self-measurements, parental measurements, and researcher measurements, as well as a strong concordance between self-/parental measurements and researcher measurements. The reliability, error probability, and concordance with researcher measurements were adequate for adult self-measurements and were particularly good for parents measuring their child’s HC. Both were within the acceptable limits even for a skilled anthropometrist [17].

The degree of intra-rater reliability of each researcher (ICC = 0.98–0.99) is comparable to previous studies looking at the reliability of adult self-measurements of other body parts [18] and the relative TEM for researcher HC measurements indicated low variability between the measurements of each researcher. The two researchers additionally showed high inter-rater reliability (ICC = 0.933) and low TEM between the means of their two measurements of the same adult participant, similar to findings by Bradley et al. [15].

Adult self- and parental measurements of HC showed strong concordance with researcher measurements and the TEM was acceptable, suggesting little variability between the measurement of HC by the adult/parent and that of the trained researcher. Indeed the reliability of adult or parent measurements, according to the ICC 95% CIs, could not be statistically differentiated from those of researchers, suggesting that self-/parent measurements of HC reflect reliable data for both research and clinical purposes. These findings are comparable to those found for self-measurement of other body parts [19] and parental measurements of child HC in this study showed similar ICC reliability to those found by Bradley et al. in 1- to 6-week-old infants [15].

It should be noted that, with regards to limitations, adults and children in this study were typically developing, so it is unclear whether these same levels of reliability would be found in clinical populations. Furthermore, it is recommended that researchers and professionals think carefully about their instructions and training if they intend to collect self-/parental HC measurements and to pilot test their approach before employing it in a large scale.

## Conclusions

To summarize, the results of this study provide strong evidence for confidence in adult self-measurements and parental measurements of HC, suggesting that trained researchers or clinicians may not always be required to obtain reliable HC measurements. This finding is applicable in paediatric, neurological, psychiatric, and psychological research, where more larger and rigorous studies might be conducted using lay measurements of HC, as well as in clinical practice.

## Abbreviations

HC: Head circumference; ICC: Intraclass correlation coefficient; CI: Confidence interval; TEM: Technical error or measurement.

## Competing interests

The authors declare that they have no competing interests.

## Authors’ contributions

JS, TT, AH, and SBC contributed to study design, JS, TT, and KA collected the data, JS and TT conducted statistical analysis, JS drafted the manuscript. All authors read and approved the final manuscript.

## Supplementary Material

Additional file 1 Head circumference instructions. Click here for file
