# Supplementary material for: Reliability of self, parental, and researcher measurements of head circumference
Source: Mol Autism. 2014 Jan 10;5:2. doi: 10.1186/2040-2392-5-2 (PMC3904212; doi:10.1186/2040-2392-5-2)
Supplement: Additional file 1 — Head circumference instructions. [file 2040-2392-5-2-S1.docx]

**Head Circumference Instructions**

- *A photograph (not included here) showed an adult male measuring his own head circumference at the desired placement (occipital-frontal perimeter)*
- Starting from approximately the front of the head, loop the measuring tape around the head:
  - Above the ears and slightly above the eyebrows
  - Try to capture the **MAXIMUM distance** around the head (i.e. where your head is the largest)
  - Pull the tape tightly, trying to get it as close to the scalp as possible
- Please report the length in centimetres, repeating the measuring procedure *twice* for the most accurate distance. The differences in head circumference we are looking for are very small, so please be as precise as you can, reporting in half centimetres if applicable (i.e. 45.5cm)
